# Affective distraction by emotional arousal during visual attention: a comparative study with young and older adults

**DOI:** 10.1007/s10339-025-01294-5

**Published:** 2025-08-12

**Authors:** José Bourbon-Teles, Pedro J. Rosa, Anna Valente, Victoria Rosa, Jorge Oliveira

**Affiliations:** 1 HEI-Lab: Digital Human-Environment Interaction Labs, Lusófona University, Lisbon, Portugal; 2 Instituto Superior Manuel Teixeira Gomes (ISMAT), Portimão, Portugal; 3 Universidade Lusófona - Centro Universitário Lisboa, 376 Campo Grande, Lisboa, 1749-024 Portugal

**Keywords:** Emotional arousal, Hedonic valence, Visual attention, Distraction, Aging

## Abstract

Irrelevant affective/emotional stimuli for a given cognitive task can interfere with visual attention. Some studies indicate that emotionally arousing stimuli can unintentionally divert attention and act as sources of distraction This study aimed to test, regardless of the valence factor, the impact of emotional arousal on attentional interference in young adults and older adults. The interference of arousal (high-arousing vs. low-arousing vs. /neutral) was examined through behavioural measures, specifically response times and response error rates. The results revealed that arousal modulates attention differently across age groups. Older adults showed a facilitation effect in the presence of low-arousal stimuli, improving their cognitive performance compared to high and neutral arousing stimuli. By comparison, no significant effects of arousal on cognitive performance were observed in young adults. These findings highlight the differential role of emotional arousal in attentional performance across the lifespan, most notably its facilitation effect in older age, and underscore the relevance of considering arousal when developing strategies to support cognitive functioning in healthy aging.

## Introduction

The study of factors that enhance distraction during focused visual attention remains an active field of research. Theories of visual attention suggest that exogenous factors, such as salient, unexpected, and task-irrelevant stimuli, can interfere with performance on visual attention tasks, while endogenous factors, such as the level of internal attention, also play a crucial role (Beck and Lavie 2005; Corbetta and Shulman 2002). Moreover, affective or emotionally-laden stimuli that are irrelevant to a given cognitive task also seem to interfere with visual attention, potentially acting as salient distractors (Heim et al. 2013; Ihssen et al. 2007).

Research in the unimodal visual domain has shown that high-arousing emotional distracting images (e.g., erotic or mutilation scenes) can disrupt visual attention tasks more than neutral images (e.g., flowers) (Heim et al. 2013; Ihssen et al. 2007). These findings reflect their potential of such stimuli to signal threatening and/or rewarding events that can automatically attract attentional resources.

Complementary findings in intermodal studies, such as those in the visuo-auditory domain, also suggest that high-arousing emotional distracting sounds may also impair visual attention (Heim et al. 2019) though some research reports conflicting results, suggesting reduced interference from highly arousing distractor sounds versus moderately arousing distractor sounds (Bonmassar et al. 2023). These mixed findings highlight the need to better isolate the specific contributions of arousal and valence to study attentional distraction.

The joint/interactive effect of valence-arousal on attentional distraction has been tested in previous studies, reporting that high-arousing positive and negative distracting stimuli impair visual attention performance to a greater extent compared to emotionally neutral stimuli (e.g., Heim et al. 2013; Ihssen et al. 2007). These findings while relevant and impactful can make it somewhat difficult to isolate the specific effects of arousal on attentional distraction from that of valence (Chan and Singhal 2015; Heim et al. 2013; Ihssen et al. 2007).

Thus, our study adopts a more targeted approach by independently manipulating arousal levels within the same valence category (positive). The main aim of the present study is to investigate how emotionally arousing stimuli affect visual attention under three distinct conditions: high arousal/positive valence, low arousal/positive valence and neutral condition, following the procedure of Anvari and colleagues (2023).

A secondary aim is to examine how these effects differ between young and older adults. Aging is associated with a range of cognitive changes, including alterations in attention and the ability to manage competing stimuli in the environment. Research has shown that older adults often experience a decline in their ability to maintain focus on tasks and resist distraction from irrelevant sensory information (Commodari and Guarnera 2008; Lee et al. 2018; Leiva et al. 2015, 2021; Parmentier and Andrés 2010). These difficulties may stem from age-related changes in executive control processes, such as inhibitory control, which are critical for filtering out distractions and prioritizing task-relevant information. Furthermore, emotional stimuli particularly those that are highly arousing are known to capture attention more effectively than neutral stimuli, potentially creating additional challenges for older adults in environments rich with emotional distractions (Gallant et al. 2020). Given these considerations, we aim to examine the effect of emotionally arousing distracting stimuli on concurrent visual attention performance in healthy aging. One hypothesis is that healthy older adults will display a more pronounced distracting effect elicited by irrelevant emotionally arousing stimuli compared to young adults. This hypothesis is grounded in prior evidence that older adults may have greater difficulty resisting distraction from high-arousing emotional stimuli during cognitive tasks (Gallant et al. 2020), likely due to the combined effects of diminished inhibitory control and the strong attentional capture properties of such stimuli.

At the same time, emotional processing in older adults appears to follow unique patterns. The “positivity effect” theory posits that older adults are more likely to focus on and process low-arousing/positive emotional information as a means of enhancing emotional well-being (Backs et al. 2005; Dolcos et al. 2014; Kappes et al. 2017; Kensinger 2008; Streubel and Kunzmann 2011). This suggests that emotional valence and arousal may interact differently with attentional processes in older adults compared to younger adults, potentially leading to distinct patterns of distraction and task performance. Thus, an alternative hypothesis to consider is that low arousing/positive stimuli may interfere with attention to a lesser degree when compared to high arousing and neutral distraction stimuli in aging. The selective focus on low-arousing stimuli may create a calming cognitive environment, thereby enhancing attention and facilitating task performance (Backs et al. 2005; Dolcos et al. 2014; Kappes et al. 2017; Kensinger 2008; Streubel and Kunzmann 2011).

Overall, this study aims to (1) isolate the effect of emotional arousal on attentional distraction by controlling for valence, and (2) explore how these effects interact with age-related differences in cognitive control. We hypothesize that high-arousing positive stimuli will impair attention more than low-arousing or neutral stimuli, particularly in older adults, while low-arousing positive stimuli may have less or beneficial effects on attention. By testing these hypotheses, this study will contribute to our understanding of the complex relationship between emotional arousal and attention in aging. The findings could have important implications for developing interventions aimed at improving cognitive performance in young and older adults, notably in environments rich in emotional stimuli.

## Methods

### Participants

A priori power analysis was conducted to determine the minimum required sample size for detecting a medium effect size (Cohen’s f^2^ = 0.15) in a two-level linear mixed model (LMM). As G*power version 3.1.9.2 does not support sample size estimation for LMMs, we followed the approach recommended by Snijders (2005), who propose a two-step method for estimating sample size in multilevel designs: (1) Compute the required sample size as if the design were a conventional mixed ANCOVA; (2) Adjust this estimate by the design effect (DE) to account for the loss of statistical power due to clustering. For a mixed ANCOVA (within-between interaction [α = 0.05, power = 0.9, 2 age groups (younger adults vs. older adults), 6 measurements: 3 (high-arousing vs. low-arousing vs. neutral) x 2 (congruent digits vs. incongruent digits), 1 covariate (years of education); correlation of repeated measures = 0.5 and a non-sphericity correction = 0.5)] a minimum sample size of 48 participants was needed. We followed the guidance of Dattalo (2008) to adjust the number of groups to account for covariates. This method compensates for the reduction in residual degrees of freedom introduced by covariates, thereby yielding a more conservative and accurate estimate of the required sample size. Specifically, for each covariate included in the model, one additional “pseudo-group” is added in the GPower input (e.g., Carvalho and Rosa 2020). After this, the DE was calculated as:

$$ {\mathrm{DE}} = {\mathrm{1}} + \left( {\bar{n} - {\text{ 1}}} \right) \times \rho , $$

wherē ${\bar{n}}$ represents the average number of repeated observations per participant (¯ ${\bar{n}}$= 6), and ρ represents the assumed intra-class correlation coefficient (ρ = 0.05). The resulting DE was 1.25. Accordingly, the adjusted required sample size was increased to 60 participants (48 × 1.25) to maintain statistical power while accounting for the dependence of observations within subjects.

The sample of this study was composed of 60 participants. Of those, 45 comprised young adult Portuguese native speakers (20 males and 25 females with a mean age of 27 years old) and 15 older adults (1 male, 14 females, age-range: 65–88 years old, mean age = 74, SD = 5,9). The young adult participants were recruited from the University campus for voluntary participation whilst the older adults were recruited from the Benfica Parish Council. We opted to exclude one participant from the study because this participant failed to understand the purpose of the task and also had outlier scores throughout. Thus, the final analyses were based on *n* = 14 older adult participants. Participants (both young and old adults) with an active psychiatric and/or neurological disorder, or a significant history of such disorders, were excluded from the study.

All participants gave their written informed consent for the study, in accordance to the Declaration of Helsinki, and approval was obtained by the Ethics Committee of the Lusófona University (Ref. CEDIC-2024-29-16).

### Stimuli and procedure

**Fig. 1:**
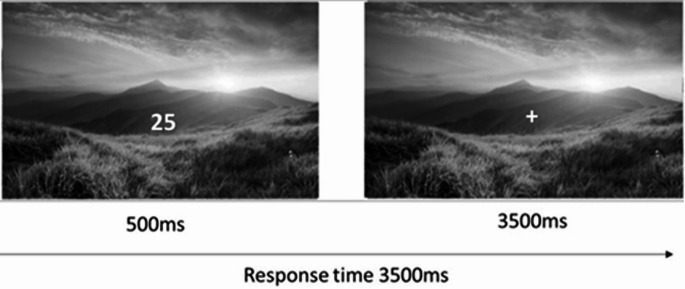
Illustrates the stimulus presentation task. The figure depicts an incongruent type of stimulus trial

The task consisted of identifying by pressing the appropriate button whether two digits displayed at the centre of the screen were congruent (if both were even or odd) and/or incongruent (if one number was even and the other was odd). In the background, an image was displayed (from three different possible types of images) that was completely irrelevant to the task and that could vary in accordance to emotional arousal (Fig. 1).

The task consisted of three distinct types of trials based on the type of distractor image: high-arousing vs. low-arousing vs. neutral. The two digits were always different, and the same combinations of digits were repeated across all the three emotional arousal conditions (high, low, and neutral arousal) to ensure that the cognitive task demands were matched. The digits were presented for 500 ms, followed by a fixation cross for 3000 ms (total response time was 3500 ms) (Fig. 1). The experimental design adopted here is a within-subjects design, and the task comprised 2 blocks of 120 trials each (with an interval period between blocks to ensure optimal attention levels) and the order of stimulus presentation was randomized. A total of 40 combinations of digits were used, half of which were congruent and the other half incongruent. Thus, 40 trials were conducted for each condition, each consisting of 20 different images presented twice: with congruent and incongruent digits (a similar experimental design was adopted by Carboni et al. 2017).

The selection of emotional stimuli/images was based on the International Affective Pictures System (IAPS) database and included high arousal/high valence (20 images), low arousal/high valence (20 images), and neutral images (Lang et al. 2008; Soares et al. 2015). The selection of stimuli following the above criteria was based on ratings of arousal and valence (i.e., on a scale from 1 to 9, from lowest to highest) by the European Portuguese community (Soares et al. 2015).

Based on a pictorial scale of nine levels (The Self-Assessment Manikin), normative values for arousal (1 = low arousal to 9 = high arousal) and valence (1 = highly unpleasant to 9 = highly pleasant) were 6.53 (arousal rating) and 7.08 (valence rating) for high arousing images (e.g., roller coaster, erotica, surfers, bungee jumping). The normative values for low arousing images (e.g., lake, sunflower, nature, field, clouds) were 3.3 (arousal rating) and 7.4 (valence rating) respectively. Finally, the normative values for neutral images (e.g., lamp, desk, hairdryer, fan) were 3.52 (arousal rating) and 4.9 (valence rating).

We have opted to specifically manipulate arousal by focusing specifically on positively valenced stimuli in order to have more valenced arousing conditions since typically negative stimuli are of high-arousing nature thereby making it difficult to include a low-arousing condition with negative stimuli (Lang et al. 2008; Soares et al. 2015).

### Statistical analysis

First, all data were inspected for outliers defined as values larger or smaller than three times the standard deviation from the mean. After, we conducted a 2-level LMM to examine the effects of arousal (high, low, neutral) and congruency (congruent, incongruent) as within-subjects factors, and age group (young, old adults) as a between-subjects factor, on the mean response times (RTs) and mean error rates. LMMs were conducted using the “lmerTest” package for R (Kuznetsova et al. 2017).Years of education was included as a covariate to control for potential confounding effects (Lövdén et al. 2020; Tun and Lachman 2008). The model included three fixed effects: age group, arousal, and congruency. Random effects consisted of a random intercept for each participant and random slopes for both congruency and arousal to account for individual differences in these effects. The analysis for the mean RTs was based on correct responses only. LMMs were fitted using restricted maximum likelihood (REML) and the degrees of freedom were computed using Satterthwaite approximations. Cohen’s d based on estimated marginal means was computed using the “emmeans” package (Lenth et al. 2018). All analyses were performed in R version 4.0.5 (R Core Team 2021) for a statistical significance level of 5%.

## Results

### Effects of age group, arousal and congruency on mean response times (RTs)

A linear mixed-effects model was used to analyze RTs as a function of Age Group, Arousal, Congruency, and Years of Education (covariate). Model fit was satisfactory, with a conditional R² = 0.924 and a marginal R² = 0.155, indicating that the fixed effects accounted for 15.5% of the variance, while the full model (including random effects) explained 92.4% of the variance in RTs as seen in Table 1.

**Table 1:** Linear mixed model analysis of mean rts: main effects and interactions with satterthwaite’s method

Effect	F	df (Num, Den)	*p*
Age Group	10.20	(1, 60.041)	0.002 **
Arousal	20.17	(2, 168.610)	< 0.001 ***
Congruency	22.20	(1, 57.061)	< 0.001 ***
Years of Education (covariate)	2.22	(1, 56.828)	0.142
Age Group × Arousal	19.63	(2, 168.610)	< 0.001 ***
Age Group × Congruency	1.05	(1, 57.061)	0.311
Arousal × Congruency	1.33	(2, 227.938)	0.266
Age Group × Arousal × Congruency	1.27	(2, 227.938)	0.283

*** *p* < .001, ** *p* < .01, * *p* < .05

**Fig. 2:**
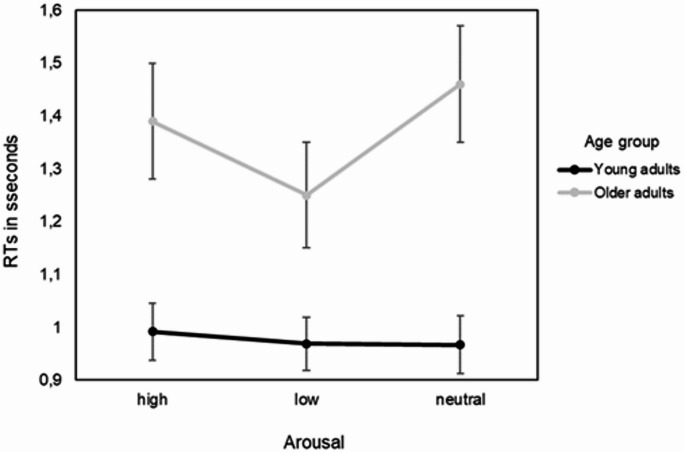
Mean RTs in seconds as a function of arousal level (high, low, neutral) and age group (young vs. older adults). Error bars represent 95% confidence intervals

Results revealed significant interaction effect Age Group × Arousal on RTs, *F*(2, 168.61) = 19.63, *p* < .001, as shown in Table 1. Simple effect analysis revealed that older adults had significantly faster RTs for low-arousing images (M = 1.25, SE = 0.10) compared to both high-arousing (M = 1.39, SE = 0.11), *p < .*001, *d* = 1.51, and neutral images (*M* = 1.46, *SE* = 0.11) *p* < .001, *d* = 2.01. Older adults also exhibited faster RTs for high arousing compared to neutral images, *p =* .026, *d* = 0.71, as depicted in Fig. 2.

We also found main effects for Age Group, *F*(1, 60.04) = 10.20, *p* = .002; Arousal, *F*(2, 168.61) = 20.17, *p* < .001; and Congruency, *F*(1, 57.06) = 22.20, *p* < .001 on the mean RTs. Incongruent trials (*M* = 1.22, *SE* = 0.06) were associated with significantly slower RTs compared to congruent trials (*M* = 1.12, *SE* = 0.05), *p* < .001, *d* = 0.89. Years of education, included as a covariate, was not significantly associated with RT (all *ps* > 0.05) (see Table 1). No other interactions or main effects were found (Table 1).

### Effects of age group, arousal and congruency on mean error rates

A linear mixed-effects model was conducted to examine the effects of Age Group, Arousal, Congruency, and Years of Education (covariate) on mean error rates. The model showed a marginal *R*² = 0.295 and a conditional *R*² = 0.929, indicating that fixed effects explained 29.5% of the variance, and the full model including random effects accounted for 92.9% as seen in Table 2.

**Table 2:** Linear mixed model analysis of mean error rates: main effects and interactions with satterthwaite’s method

Effect	F	df (Num, Den)	*p*
Age Group	0.0034	(1, 56.571)	0.954
Arousal	0.3750	(2, 207.909)	0.688
Congruency	3.788	(1, 56.991)	0.057 .
Years of Education	21.046	(1, 56.288)	< 0.001 ***
Age Group × Arousal	3.588	(2, 207.909)	0.029 *
Age Group × Congruency	11.452	(1, 56.991)	0.001 **
Arousal × Congruency	1.146	(2, 227.910)	0.320
Age Group × Arousal × Congruency	0.340	(2, 227.910)	0.712

**Fig. 3:**
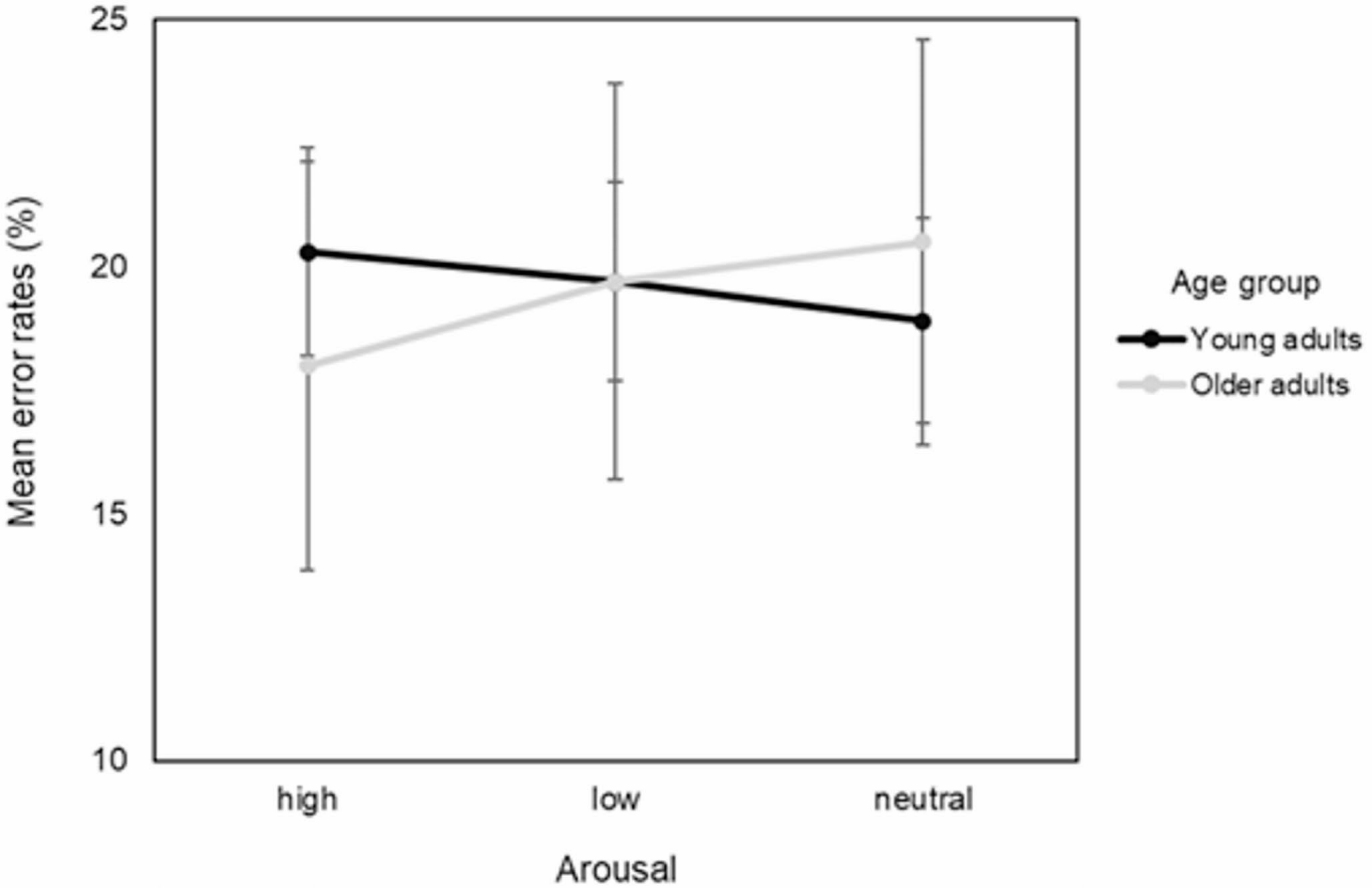
Mean error rates as a function of arousal level (high, low, neutral) and age group (young vs. older adults). Error bars represent 95% confidence intervals

Results indicated significant interactions for Age Group × Arousal, *F*(2, 207.91) = 3.59, *p* = .029 on mean error rates as seen in Fig. 3.

However, when examining the simple effects analyses, we found no statistically significant differences in arousal conditions. For older adults, error rate for high-arousing images (*M* = 18.0, *SE* = 4.14) did not differ significantly from low-arousing (*M* = 19.7, *SE* = 4.00), *p* = .568, *d* = 0.36, or neutral images (*M* = 20.5, *SE* = 4.11), *p* = .154, *d* = 0.53. Similarly, low-arousing and neutral images did not differ significantly, *p* > .999, *d* = 0.17. For young adults, error rates for high-arousing images (*M* = 20.3, *SE* = 2.10) was also not significantly different from low-arousing images (*M* = 19.7, *SE* = 2.01), *p* > .999, *d* = 0.13, or neutral images (*M* = 18.9, *SE* = 2.07), *p* = .156, *d* = 0.29, nor did low-arousing and neutral images differ significantly, *p* = .841, *d* = 0.16.

**Fig. 4:**
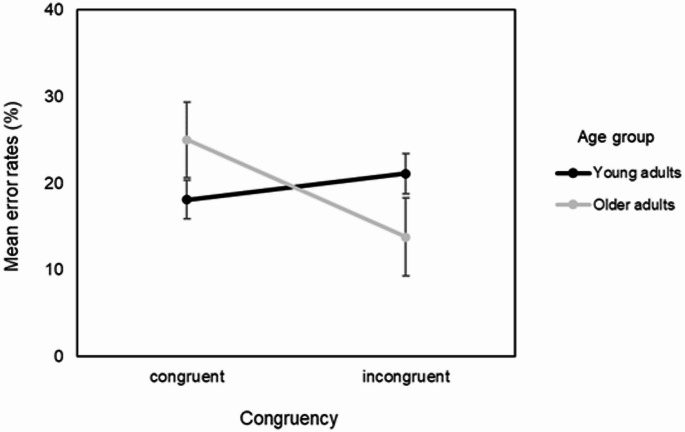
Mean error rates as a function of congruency (congruent vs. incongruent) and age group (young vs. older adults). Error bars represent 95% confidence intervals

There was also an Age Group × Congruency interaction effect on mean error rates *F*(1, 56.99) = 11.45, *p* = .001. Simple effects analyses revealed that older adults presented significantly higher error rates for congruent stimuli (*M* = 25.0, *SE* = 4.35) than incongruent stimuli (*M* = 13.8, *SE* = 4.48), *p* = .004, *d* = 0.80, indicating a large effect size. In contrast, young adults showed no significant difference between congruent (*M* = 18.1, *SE* = 2.22) and incongruent stimuli (*M* = 21.1, *SE* = 2.30), *p* = .146, *d* = 0.38. These results suggest that stimulus congruency impacted older adults’ ratings more strongly than young adults’, as shown in Fig. 4.

Years of education was a significant covariate in predicting the error rate, *b* = -2.08, SE = 0.45, *t*(56.29) = -4.59, *p* < .001, indicating that higher levels of education were associated with significantly lower error rates. No other significant effects (simple and interaction) were found (all ps > 0.05) (see Table 2).

## Discussion

The purpose of this study was to examine the distraction effects by emotional arousal on visual attention across different age groups, notably young and old adults.

The findings showed there was an interaction effect between the age and arousal on cognitive performance. The older adults exhibited faster RTs in the low-arousal condition relative to both high-arousal and neutral conditions. Thus, this pattern may reflect the calming effect that low-arousal images have on older adults, which could enhance their focus and reduce cognitive interference (Backs et al. 2005; Dolcos et al. 2014; Kappes et al. 2017; Kensinger 2008; Streubel and Kunzmann 2011). Older adults often experience greater difficulty with attentional control, particularly in the presence of emotionally intense stimuli (Gallant et al. 2020). The low-arousal images may have provided a more relaxed cognitive environment, allowing them to allocate their attention more effectively to the task. Interestingly, older adults exhibited faster RTs in the high-arousal condition compared to the neutral condition. This could perhaps indicate that high-arousal stimuli, despite being distracting, elicit a quicker emotional or cognitive response. Older adults may rapidly disengage from these stimuli to maintain focus on the task. This behaviour aligns with theories such as the positivity effect, which suggest that older adults prioritize emotional regulation by downregulating negative or intense emotions (e.g., Kappes et al. 2017).

By comparison, no significant effect of arousal on cognitive performance was observed in the young adults. While visual inspection of numerical RTs across arousal conditions indicates a slowing of responses in the high arousing condition compared to the remaining arousing conditions these differences were not statistically significant and thus remain inconclusive. Consequently, the current findings do not support previous claims that highly arousing or emotionally salient stimuli interfere with cognitive processes by prioritizing affective over task-relevant information (Vuilleumier 2005; Wessa et al. 2013; Zsidó 2024; Mather and Sutherland 2011). One possible explanation for the absence of an effect is that any influence of arousal on attentional performance may be subtle, variable across individuals, and require a larger sample size to detect reliably (Goodhew and Edwards, 2009; Lee et al. 2014; Most et al. 2005).

Thus, it seems reasonable to conclude that emotional arousal affects attentional performance, particularly in older adults; while any specific effect it may have on young adults still remains elusive, at least within the scope of this study.

While it could be expected that high-arousal stimuli would exacerbate the congruency effect by impairing inhibitory control and, conversely, that low-arousal stimuli might reduce it, no significant interaction between arousal and congruency was observed. This may suggest that the attentional mechanisms underlying general task performance (e.g., RTs) and those involved in conflict resolution during incongruent trials are partly independent. Alternatively, the absence of an interaction could reflect limited statistical power or subtle effects that require more sensitive experimental designs to detect (Goodhew and Edwards 2009; Lee et al. 2014; Zsidó 2024).

Although simple effects analyses did not reveal statistically significant differences, the interaction pattern in mean error rates suggests opposing trends in how emotional arousal influences attentional performance across age groups. Young adults showed slightly higher error rates in the high arousal condition, potentially reflecting mild susceptibility to emotional distraction (Zsidó 2024). However, it is important to reiterate that these results are purely numerical and remain inconclusive (as in the case of the RT data). In contrast, older adults exhibited their lowest error rates in the high arousal condition and the highest in the neutral condition. This pattern, while not statistically significant, may reflect age-related differences in emotional processing and regulation. Older adults are thought to engage more actively in emotion regulation, potentially allowing them to disengage from emotionally charged stimuli and refocus more effectively on task-relevant stimuli (Kappes et al. 2017). Neutral stimuli, by contrast, may lack emotional depth that aids engagement, thereby increasing cognitive load and error rates. Although preliminary, these findings may indicate meaningful age-related differences and highlight the need for further research with larger samples.

The interaction effects between age and task congruency on mean error rates is also of interest. Even though non-significant, the young adults exhibited higher error rates in the incongruent condition compared to the congruent condition, which may reflect additional cognitive demands of resolving interference in incongruent trials. Older adults, however, displayed the opposite pattern, with significantly higher error rates in the congruent condition. This could indicate a strategic shift in cognitive processing among older adults, whereby they prioritize attentional resources toward resolving more demanding trial conditions (i.e., incongruent trials). This compensatory strategy may reflect an adaptive mechanism to maintain overall performance in the face of age-related declines in cognitive flexibility, processing speed and conflict resolution (Langner et al. 2015; Park and Reuter-Lorenz 2009; Reuter-Lorenz and Cappell 2008).

One limitation of the present study is the imbalance in group sizes between younger and older participants (45 vs.15) respectively. Although the use of linear mixed models mitigates some concerns, as they are robust to unequal group sizes, this discrepancy may raise concerns about statistical power, especially for interactions. This group imbalance may have contributed to the limited power in detecting significant comparisons within the smaller older adult group. Smaller sample sizes reduce the precision of estimates and the ability to detect true effects, especially when using conservative corrections (e.g. Bonferroni). Thus, while the overall interaction effect was significant, the limited power in subgroups, particularly the older adults, likely prevented significant pairwise differences from emerging. Future studies should aim for more balanced groups to strengthen the reliability of between-group comparisons. Furthermore, future studies should aim to capture the heterogeneity within the ageing population, considering individual differences in cognitive, emotional, and health-related factors that may influence performance.

In addition, while the study examined arousal effects, all emotional stimuli were of positive valence. This limits the ability to generalize findings to other emotional contexts, particularly negative valence stimuli, which may elicit different patterns of distraction or facilitation. Negative high-arousal stimuli, such as threatening or distressing images, could evoke stronger attentional biases, especially in younger adults, and may interact differently with age-related mechanisms of emotional regulation. However, as mentioned earlier this strategy can also pose significant challenges since typically negative stimuli are of high-arousing nature thereby making it difficult to include a low-arousing condition with negative stimuli (Lang et al. 2008; Soares et al. 2015).

Finally, while our design aimed to manipulate arousal while keeping valence relatively constant by using positively valenced stimuli across conditions, normative ratings indicated a slight difference in valence between high and low arousing images (M_high = 7.08, M_low = 7.40). Although both sets of stimuli were clearly positive in valence, this variation may have partially contributed to the observed effects, particularly among older adults, who are known to preferentially process positively valenced information. However, given that the valence difference was modest and both conditions remained within the positive range, we believe that the primary effects are more likely driven by arousal, with possible additive contributions from valence. Nevertheless, future research may benefit from more tightly controlling or modeling these dimensions to better isolate their unique roles in attentional processing across age groups.

Together, these findings provide important insights into the effects of emotional arousal on concurrent visual attention across age groups. While older adults seem to benefit from the facilitation provided by low-arousal conditions, the impact of emotional arousal on attention performance in young adults still remains elusive, and warrants further investigation. These age-related patterns highlight the need to consider both arousal and emotional valence when designing cognitive interventions. Tailoring emotional environments to match age-specific cognitive and affective needs may help optimize attentional performance across the lifespan.

## Data Availability

The data that support the findings of this study are openly available in OSF at https://osf.io/n6u7d/.
